# Treatment of infected tibial nonunion with angular deformity and shortening using the Masquelet technique combined with the Ilizarov technique: A case report

**DOI:** 10.1016/j.tcr.2026.101335

**Published:** 2026-04-22

**Authors:** Akifumi Honda, Yoshinobu Watanabe, Gen Sasaki, Natsumi Saka, Mari Nishizawa, Kunihiko Arakawa, Hirotaka Kawano

**Affiliations:** a Trauma and Reconstruction Center, Teikyo University Hospital, 2-11-1 Kaga, Itabashi-ku, Tokyo, 173-8606, Japan; b Department of Orthopaedic Surgery, Teikyo University School of Medicine, 2-11-1 Kaga, Itabashi-ku, Tokyo, 173-8605, Japan

**Keywords:** Induced membrane technique, Nonunion, Bone defect, Tibial fracture, Ilizarov technique, Soft tissue lengthening

## Abstract

The induced membrane technique (Masquelet technique) combined with the Ilizarov technique may provide a novel strategy for managing infected nonunion with angular deformity and limb shortening within a relatively short treatment period. We report a 45-year-old male with a Gustilo–Anderson type IIIA open tibial fracture complicated by fracture-related infection, 33° valgus deformity, and 50-mm limb shortening. Treatment was performed in three stages: radical debridement with rapid soft-tissue lengthening at 3 mm/day using an Ilizarov external fixator; definitive fixation with an intramedullary nail and Masquelet technique; and cancellous bone grafting with beta-tricalcium phosphate, delayed to 10 months after the second stage due to psychiatric comorbidity. Bone healing was achieved uneventfully within 2 years, with a residual limb-length discrepancy of <1 cm and good functional recovery. This case illustrates that rapid soft-tissue lengthening can effectively correct limb shortening and malalignment, thereby reducing the duration of external fixation and its complications. Moreover, delayed bone grafting did not compromise bone healing, underscoring the robustness of the Masquelet technique combined with intramedullary nail fixation. Overall, this combined approach represents a promising strategy for complex infected nonunions, reducing patient burden while ensuring reliable bone reconstruction and functional recovery.

## Highlights

• Rapid soft tissue lengthening at 3 mm/day corrected a 50-mm tibial shortening.
• The Masquelet technique with IM nail succeeded after deformity correction.
• External fixator use was limited to the soft tissue distraction phase only.
• Bone grafting delayed by 10 months still achieved successful bone healing.
• This combined method offers reduced patient burden and good functional recovery.

## Introduction

The induced membrane technique, also known as the Masquelet technique, has been reported as a useful method for reconstructing bone defects due to infected nonunion. Favorable clinical outcomes have been described in cases of infected tibial nonunion treated with the Masquelet technique combined with intramedullary nail fixation [1], [2], [3], [4]. However, this technique is generally contraindicated in the presence of associated limb shortening or angular deformity.

Conversely, the Ilizarov technique can simultaneously address the bone defects, angular deformities, and limb shortening [5], [6], [7]. To achieve adequate bone formation, however, the rate of bone transport is usually limited to 0.5–1 mm/day [8], [9]. Consequently, this method requires prolonged application of an external fixator, which is frequently associated with complications such as pin site infection, muscle contracture, and patient discomfort [10], [11], [12].

To reduce patient morbidity, combining the Masquelet and Ilizarov techniques, performing rapid soft-tissue lengthening with an external fixator prior to Masquelet treatment, may significantly shorten the duration of external fixation. To our knowledge, no such approach has been previously reported. Here, we present a case of infected tibial nonunion with severe and functionally unacceptable angular deformity and limb shortening, successfully managed by rapid soft-tissue lengthening using an external fixator, followed by the Masquelet technique with intramedullary nail fixation.

## Case report

**Fig. 1:**
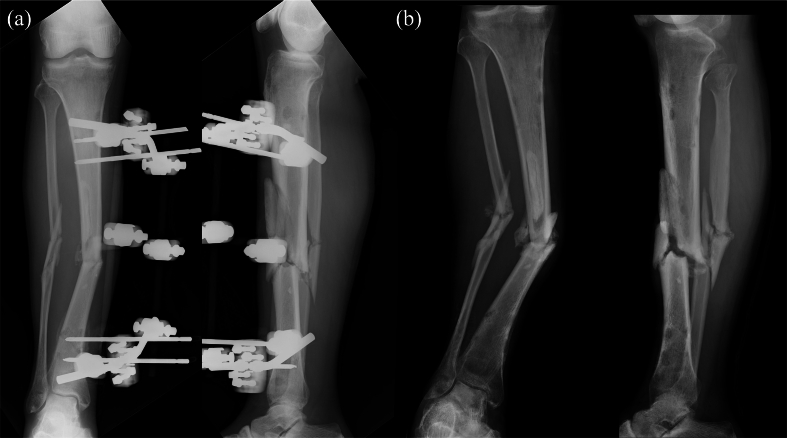
Radiographic images (a) immediately following the application of the external fixator and (b) at the time of the first visit to our department.

A 45-year-old male sustained a Gustilo–Anderson classification type IIIA open tibial and fibular fracture of the right leg after a motorcycle collision with a car. On the day of injury, he underwent debridement and irrigation, and on the following day, definitive fixation with a Hoffman external fixator (Stryker, Portage, MI, USA) was performed (Fig. 1a). At 3 months post-injury, he developed skin necrosis, fistula formation, and symptomatic pin site infection. The patient was diagnosed with a fracture-related infection, and the external fixator was removed. Methicillin-resistant *Staphylococcus aureus* was identified as the causative organism. He was subsequently referred to our hospital for radical management of infected tibial nonunion. His past medical history was unremarkable, although he had a psychiatric history of schizophrenia, treated with antidepressants and hypnotics.

**Fig. 2:**
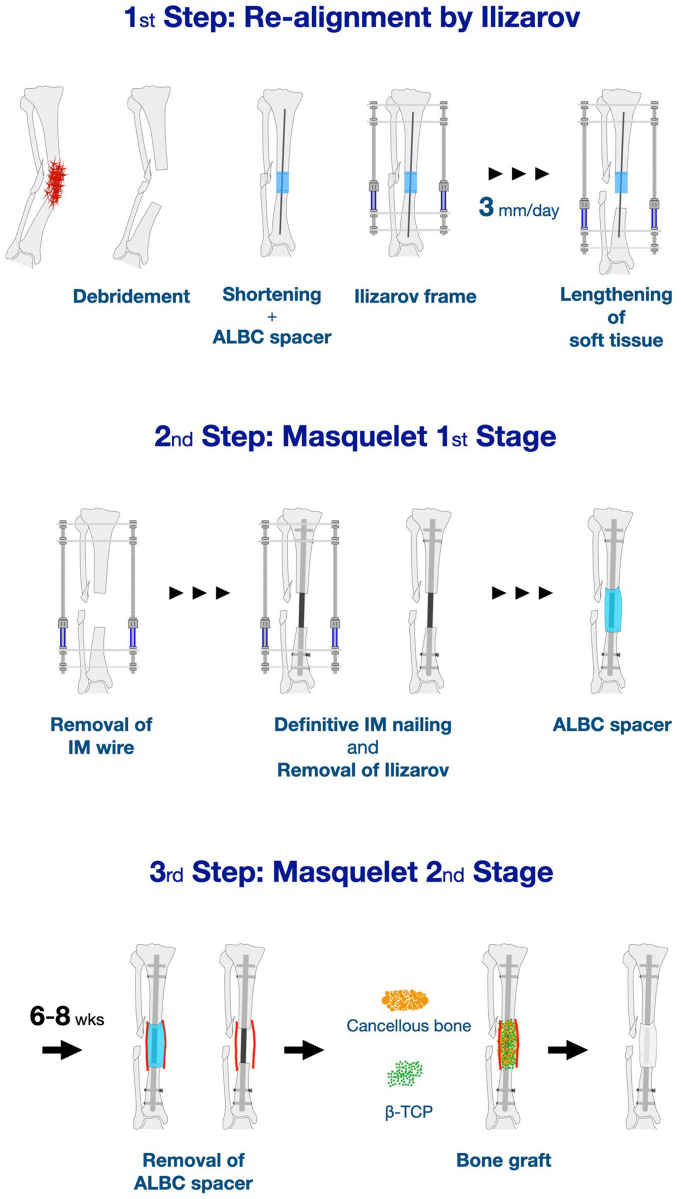
Schematic diagram of the treatment strategy using the Ilizarov and Masquelet techniques.

At presentation, the right lower leg showed no abnormal findings except for pigmentation, and all fistulae had closed. Radiographs demonstrated nonhealing tibial and fibular fractures, and a 33° valgus deformity and a 50-mm limb shortening (Fig. 1b). A three-step treatment strategy was planned (Fig. 2).

**Fig. 3:**
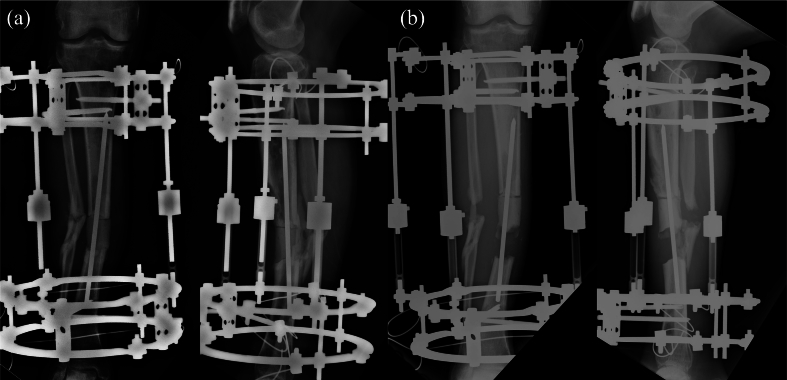
Radiographic images during (a) application of the Ilizarov external fixator following angular deformity realignment and (b) soft tissue lengthening at a rate of 3 mm/day.

The first step was radical debridement of the infected area and application of an Ilizarov external fixator (Smith & Nephew Richards, Memphis, TN, USA) to perform soft tissue lengthening. The main bone fragments were aligned as much as possible, except for shortening (Fig. 3a). An intramedullary wire was inserted across the fragments, and the dead space was filled with an antibiotic-loaded bone cement spacer, prepared by mixing vancomycin powder (2 g per 40 g of cement) with bone cement. Postoperatively, the lower leg was lengthened at a rate of 3 mm/day until it matched the contralateral side (Fig. 3b).

**Fig. 4:**
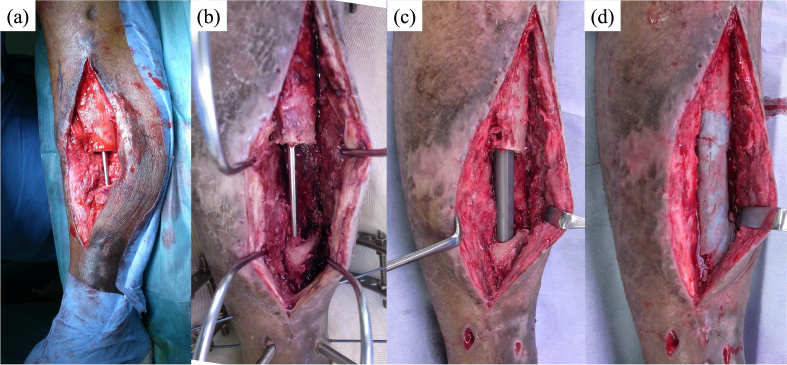
Intraoperative findings (a) The main bone fragment is temporarily fixed by an intramedullary wire just before mounting the antibiotic-laden bone cement (ALBC) spacer during the first stage; (b) the ALBC spacer is removed, and a definitive intramedullary locked nail is applied during the second stage; (c) the bone gap is filled with the ALBC spacer.

In the second step, the external fixator was removed, and the tibia was definitively stabilized with an intramedullary locked nail (Fig. 4a-c). The bone defect was filled with an antibiotic-loaded bone cement spacer containing vancomycin (Fig. 4d), corresponding to the first stage of the conventional Masquelet technique.

**Fig. 5:**
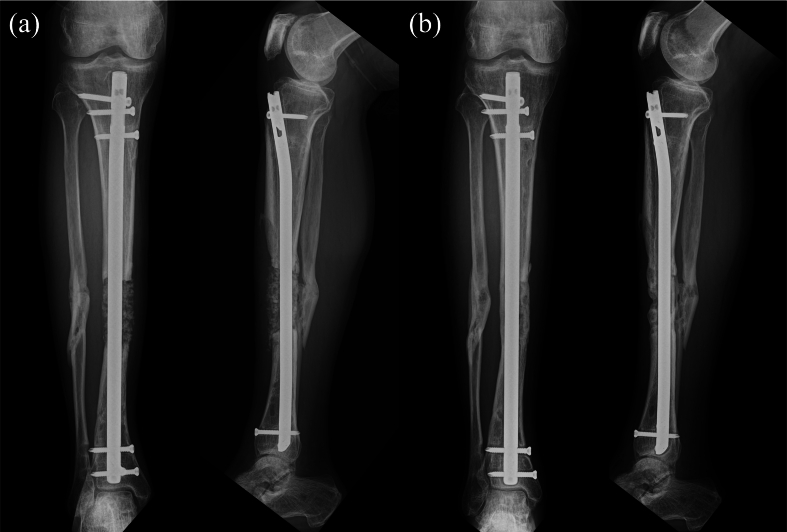
Radiographic images (a) immediately following cancellous bone and beta-tricalcium phosphate grafting and (b) at 10 months after.

Cancellous bone graft was originally planned for 6 weeks after the second surgery. However, due to worsening psychiatric symptoms, the patient required admission to a psychiatric hospital, and grafting was delayed until 10 months postoperatively. During this period, he was able to ambulate with full weight bearing using crutches, supported by the intramedullary nail and cement spacer. At 10 months, autologous cancellous bone harvested from the iliac crest and mixed with beta-tricalcium phosphate (β-TCP) was used for grafting (Fig. 5a). During the second-stage surgery, intraoperative Gram staining and bacterial cultures were performed, and no bacteria were detected. Partial and full weight bearing were permitted at 1 and 4 months after grafting, respectively. Pharmacological thromboprophylaxis was not administered throughout the treatment course because early mobilization and full weight bearing were possible. Radiographs showed uneventful healing, and union was achieved by 10 months post grafting (Fig. 5b). Final standing full-length limb radiographs revealed a limb length discrepancy of <1 cm. At the 2-year follow-up, the patient was walking independently with full weight bearing, without assistive devices, with no limitation of knee motion and > 80% of the ankle motion compared with the contralateral side.

## Discussion

This case provides two important clinical insights. First, soft tissue lengthening at a rate of 3 mm/day was effective for correcting limb shortening. Second, once limb-length discrepancy and malalignment were corrected, the bone defect at the nonunion site could be reconstructed using the conventional Masquelet technique combined with intramedullary nailing.

Limb shortening can be corrected by soft tissue lengthening using an external fixator [5], [6], [7], [10]. When reconstructing large bone defects after radical debridement of infected nonunion using the conventional Ilizarov method, the limb lengthening rate is generally limited to 0.5–1 mm/day to achieve adequate bone formation with the bone transport technique [8], [9]. In the present case, reconstructing a 60-mm bone defect solely by bone transport would have required 2–4 months, and prolonged use of the external fixator during consolidation might have extended the total fixation period to approximately 1 year [10], [13]. In contrast, our technique utilized the Ilizarov external fixator only for correcting limb-length discrepancy. The required 40-mm soft tissue lengthening was achieved at 3 mm/day, eliminating the need for a waiting period. Approximately 2 weeks after the initial surgery, bone reconstruction was initiated with the Masquelet technique, thereby reducing the duration of external fixation and patient discomfort.

After correction of limb length and alignment, bone reconstruction was accomplished using the standard two-stage Masquelet technique with an intramedullary nail. Although the interval between stages was extended to 10 months due to psychiatric comorbidity, bone formation and healing occurred as expected after bone grafting. Masquelet et al. recommended an optimal interval of 4-8 weeks between the first and the second stage [2], [3]. As a routine practice, we often perform cancellous bone grafting with β-tricalcium phosphate (β-TCP) at an approximate 50:50 volume ratio about 6 weeks after the first stage [14]. In this case, despite the prolonged delay, bone healing was comparable to conventional outcomes. Because the intramedullary nail and cement spacer maintained sufficient structural stability, the patient's activities of daily living and quality of life were preserved during psychiatric treatment. Furthermore, combining β-TCP with autologous cancellous bone for volume augmentation proved effective even after 10 months of spacer placement.

No recurrence of infection was observed during the two-year follow-up period.

In conclusion, for infected nonunion with angular deformity and/or limb shortening, rapid soft-tissue lengthening with an external fixator after debridement, followed by the Masquelet technique with intramedullary nailing, represents a promising alternative therapeutic strategy.

## CRediT authorship contribution statement

**Akifumi Honda:** Writing – original draft, Investigation, Data curation, Conceptualization. **Yoshinobu Watanabe:** Writing – review & editing, Visualization, Supervision, Investigation, Conceptualization. **Gen Sasaki:** Writing – review & editing, Validation, Investigation. **Natsumi Saka:** Writing – review & editing, Validation, Investigation. **Mari Nishizawa:** Writing – review & editing, Validation, Investigation. **Kunihiko Arakawa:** Writing – review & editing, Visualization, Validation, Investigation. **Hirotaka Kawano:** Writing – review & editing, Supervision, Investigation.

## Ethical statement

Informed consent for publication was obtained from the patient, and this report was conducted in compliance with the tenets of the Declaration of Helsinki.

## Declaration of Generative AI and AI-assisted technologies in the writing process

During the preparation of this work, the authors used ChatGPT (OpenAI) to improve the clarity and readability of the manuscript. After using this tool, the authors reviewed and edited the content as needed and take full responsibility for the content of the published article.

## Declaration of competing interest

The authors declare the following financial interests/personal relationships which may be considered as potential competing interests: Akifumi Honda, Yoshinobu Watanabe, Gen Sasaki, Natsumi Saka, Mari Nishizawa, Kunihiko Arakawa, Hirotaka Kawano reports a relationship with Zimmer Biomet GK that includes: speaking and lecture fees. Akifumi Honda, Yoshinobu Watanabe, Gen Sasaki, Natsumi Saka, Mari Nishizawa, Kunihiko Arakawa, Hirotaka Kawano reports a relationship with Stryker Corporation that includes: speaking and lecture fees. Yoshinobu Watanabe, Hirotaka Kawano reports a relationship with Asahi Kasei Pharma Corporation that includes: speaking and lecture fees. Yoshinobu Watanabe, Hirotaka Kawano reports a relationship with Teijin Pharma Limited that includes: speaking and lecture fees. If there are other authors, they declare that they have no known competing financial interests or personal relationships that could have appeared to influence the work reported in this paper.
